# Health Perceptions, Self and Body Image, Physical Activity and Nutrition among Undergraduate Students in Israel

**DOI:** 10.1371/journal.pone.0058543

**Published:** 2013-03-14

**Authors:** Liat Korn, Ester Gonen, Yael Shaked, Moria Golan

**Affiliations:** 1 Ariel University Center of Samaria, School of Health Sciences, Department of Health Management, Ariel, Israel; 2 Ariel University Center of Samaria, School of Health Sciences, Department of Nutrition, Ariel, Israel; 3 Tel Hai Academic College, Department of Nutrition, Upper Galilee, Israel; Edinburgh University, United Kingdom

## Abstract

**Purpose:**

This study examines health perceptions, self and body image, physical exercise and nutrition among undergraduate students.

**Methods:**

A structured, self-reported questionnaire was administered to more than 1500 students at a large academic institute in Israel. The study population was heterogenic in both gender and fields of academic study.

**Results:**

High correlations between health perceptions, appropriate nutrition, and positive self and body image were found. The relationships between these variables differed between the subpopulation in the sample and the different genders. Engagement in physical exercise contributed to positive body image and positive health perceptions more than engagement in healthy nutrition. Nutrition students reported higher frequencies of positive health perceptions, positive self and body image and higher engagement in physical exercise in comparison to all other students in the sample.

**Conclusions:**

This study suggests, as have many before, that successful health promotion policy should reflect a collectivist rather than an individualist ethos by providing health prerequisites through a public policy of health-promotion, where the academic settings support a healthy lifestyle policy, by increasing availability of a healthy, nutritious and varied menu in the cafeterias, and offering students various activities that enhance healthy eating and exercise.

**Implications and contribution:**

This study examined health perceptions, self-image, physical exercise and nutrition among undergraduate students and found high correlations between these topics. Nutrition students reported higher frequencies of positive health perceptions, and positive self and body image and engaged more in physical exercise when compared with all other students in the sample.

## Introduction

A healthy lifestyle, proper nutrition and physical activity have been found to be protective factors against many diseases, including cardiometabolic diseases, hypertension, various cancers, obesity and related diseases [1]. A healthy lifestyle among adolescents and young adults leads to several psychosocial and behavioral protective factors as well as risk factors [2]. While risk factors raise the probability of involvement in risk behaviors, protective factors reduce the probability of involvement in risk behaviors by providing a model of positive social behaviors, by means of social and personal supervision and control, as well as a supportive social environment. Both risk and protective factors are present in all of our social and personal systems [2]. Obtaining knowledge on how and why to employ a healthy lifestyle is a basic developmental need and an important tool for managing challenges arising from the current “obesity epidemic” [3]–[5]. It is important to note that health behaviors are formulated throughout life with great emphasis on the earlier stages of life [3].

Individuals who wish to pursue higher education tend to do so during their late adolescents and young adulthood years. Variables innate in the college experience make this environment a potentially significant risk factor for the instigation of disordered eating [6], [7]. Physiological and psychological factors in the college environment, which may cause the onset of disordered eating include: identity and role changes, insufficient exercise, cafeteria food, and the availability and the ease of snacking on junk food [7], [8]. Thus, as health behaviors continue to form during the crucial years spent in pursuing higher education, interventions at this stage may have a lasting impact on health promotion and disease prevention [9].

In their qualitative study, based on eight university focus groups, Greaney et al., [10] found that college students identified temptations, lack of self discipline, and social and environmental issues such as time constraints and willingness to eat unhealthy food as obstacles to keeping a healthy lifestyle or maintaining their weight. Furthermore, Maglione & Hayman [11] reported a positive correlation between social support, self-efficiency and a commitment to engage in physical activity among under-privileged college students, as they demonstrated that those with high levels of social support, self efficiency and a commitment to plan for physical activity reported higher physical activity performance.

In their examination of knowledge, beliefs and attitudes about weight control and eating disorders among trainee home economics and physical education teachers, O'Dea & Abraham [12] found that 20% of females and 13% of males regularly skipped breakfast. Furthermore, participants were found to lack knowledge about weight control, adolescent nutritional needs and fad diets, and held misconceptions about eating disorders, with 14–72% answering questions on the subject incorrectly. The authors concluded that trainee home economics and physical education teachers require more specific training about nutrition, eating disorders, weight control, and suitable tools to better advise overweight students.

In addition, psychological and physical factors, such as negative emotions and body dissatisfaction, are predictive risk factors for disordered eating in adolescent girls, and environmental stimuli may heighten susceptibility towards eating disorders in girls with these characteristics [7]. As disordered eating behaviors and negative views of weight have been shown to be established prior to the commencement of college [13], [14], the stressful college setting may inflate pre-existing unhealthy eating habits.

Later in life, negative self image is more common among young females than males [15] and women typically report higher situational body dissatisfaction and exercising for appearance-related reasons compared to men [16], [17]. Moreover, weight has been found to be a much greater issue for women [18], who felt more overweight, dieted more, expressed more body consciousness, and reported that weight interfered more with social activities compared to men [19]. Male students have been found to be less concerned with weight and to use fewer strategies for controlling weight compared to females [20]; Furthermore, female students have been shown to have healthier habits related to alcohol consumption and nutrition but experienced more stress, while males showed higher levels of overweight and obesity and less interest in nutritional advice and health enhancing activities [21].

Stress and depression have also been found to impact health behaviors and dietary quality [22]. A study which examined medical students and students of other health disciplines found that stress manifests physically, behaviorally and emotionally due to extensive academic requirements, personality traits such as perfectionism, and additional difficulties related to student life [23], [24]. A history of weight change and weight gain has been attributed to stress and mental instability, and the inability to cope with stress and weight gain among university students and staff, have also been correlated [25].

Indeed, the benefits of physical activity and its positive influence on health, emotional well-being [26], and self esteem [26], [27] are well supported. Students who regularly engage in physical activity have higher self-esteem, report an improved external appearance, are less preoccupied with body measurements, receive more positive feedback from social surroundings and are significantly healthier, both physically and emotionally, than non-active students [26]. Furthermore, it is well documented that body image affects self-esteem [28]–[30] since a person’s weight is highly visible and affects initial impressions on others [28]. One study, which examined 1,217 college students, found that stockier students, especially women, reported a significantly higher incidence of negative self-image [29], and a related study found that the most significant factors for predicting manifestation of eating disorder symptoms are low self appraisal and dissatisfaction regarding body image [30].

One way to encourage positive health practices is through community settings. A study conducted in three American universities concluded that the physical education departments in higher education facilities should model healthy lifestyles through community activities, both in schools and in other facilities, in order to promote overall wellness including regular physical activity, good nutrition and positive health practices [31]. Kicklighter et al [32] found that students reported increased knowledge of food portions, eating a healthier breakfast and making better snacking choices, as well as exhibiting a desire for information to integrate into a healthier lifestyle, in response to community nutrition modules.

The aim of this study is to examine the effect of different variables on undergraduate students, and through this to propose appropriate interventions for improving positive health behaviors and reducing risk behaviors among this population.

Our study goals are to evaluate health perceptions, self and body image, and physical activity and nutritional habits among undergraduate students, as well as to explore the impact of gender on these variables and to study the associations between them among various academic disciplines. We hypothesize that:

1. Female students demonstrate a lower self and body image than males and engage in less physical exercise, yet keep a sensitive diet in higher frequency than male students.
2. Positive relationships will be observed between engaging in physical exercise, healthy nutrition a positive self image and health perceptions.
3. Nutrition and physiotherapy students will report healthier nutritional habits and more physical exercise compared with students from other disciplines.

## Methods

### Questionnaire

A structured, self-reported, anonymous questionnaire was distributed to undergraduate students in a large academic institute in Israel. Most of the questions originated from Jessor's Survey of *Personal and Social Development* [33], and were culturally adapted to suit the local student population. Detailed descriptions of the methodology of the survey have been previously published, including information regarding the questionnaire development and methodological assets (http://www.colorado.edu/ibs/jessor/questionnaires.html).

The questionnaire included socio-demographic parameters, questions about self perception, health perceptions, self and body image, emotional stressors, social support, social relationships, risk behaviors, nutrition and physical activity habits and psycho-somatic questions. In order to shorten the questionnaire and reduce respondent burden, it was divided and distributed in two rows, both containing core questions on every topic. Row A had expanded questions on risk behaviors, and row B had expanded questions on risk factors.

### Participants

The sample included 1574 undergraduate students (1010 females and 564 males) from the faculties of Health Sciences, Natural Sciences and Social Sciences in a large academic institute in Israel, during April–May 2009. The response rate was 93.5%. A total of 768 students completed row A of the questionnaire, 770 completed row B, and 36 students completed the pilot questionnaire. The respondent mean age was 27 (SD = 6.6, Range = 48).

### Procedures

Ethics Committee approval from the Ariel University Center was obtained prior to the pilot phase. Questionnaires were distributed within the classes, ten minutes before the end of the lesson. The survey team read an introduction before distributing the questionnaire and allowed students the opportunity to decline participation. In total, the survey team entered approximately 70 classrooms during April–May 2009.

### Measures

#### Health perceptions

Health perceptions were evaluated via four questions: 1. “How important is it to for you to feel in good shape?” 2. “How important is it for you to feel like you have plenty of energy?” 3. “How important is it for you to keep yourself in good health all year round?” 4. “How important is it for you to keep yourself fit even if it takes some extra effort?” Values ranged from 1- “not too important” to 5- “very important”. Question 5 asked “how is your health compared to others your age?” and the values ranged from 1- “my health is much worse” to 5- “my health is much better”. An index was created for health perceptions (5 items; Cronbach Alpha = .79).

#### Self image

Four items assessed self image: 1. Decision-making about important things in life 2. The ability to do schoolwork well 3. Handling setbacks and disappointments 4. Overall satisfaction with oneself. Values ranged from 1- “very good” to 4- “not so good”. An index was created for self image (4 items; Cronbach Alpha = .62).

#### Body image

Four items appraised body image: 1. Thinking you are physically attractive to other people (values ranged from 1- “very attractive” to 4- “not attractive at all”). 2. Feeling you are in better, worse or about the same physical shape compared to others your age (values ranged from 1- “much worse physical shape” to 5- “much better physical shape”). 3. Feelings about the way you look (values ranged from 1- “very satisfied” to 4- “not satisfied at all”). 4. Feelings about your weight (values ranged from 1- “I would like to lose more than 10 kilos” to 5- “I would like to gain more than 10 kilos”. An index was created for body image (first 3 items; Cronbach Alpha = .57). Item 4 was dismissed from the index.

#### Physical exercise

This item asked about the frequency of engaging in physical exercise (running, riding a bike or lifting weights). Values ranged from 1- “not at all” to 6- “more than 15 hours a week.” Additional coding or odds ratio calculation: Physical exercise was dichotomized to 1. Non active students at all even not an hour a week (value 1) and 2. Active student (values 2–6) from one hour a week to over 15 hours a week.

#### Nutrition

Of the eight items investigated pertaining to nutrition, six dealt with how much attention the respondents paid to the following: 1. Seeing that your diet is healthy. 2. Keeping down the amount of fat you eat. 3. Eating some fresh vegetables every day. 4. Eating healthy even when eating out. 5. Eating healthy snacks like fruit instead of candy. 6. Eating foods that are baked and broiled rather than fried. In answering these questions, respondents were asked to consider their regular eating habits and mark them on scale ranging from 1- “none” to 3- “a lot.” Two additional questions pertained to attention paid to these nutritional habits were asked: 7. Do you usually snack instead of eating regular meals? and 8. How often do you skip breakfast? Values ranged from 1 - almost never to 3 - most of the time. A nutrition index was built from all eight items (Cronbach Alpha = .85).

### Data analysis

Analyses included descriptive, relationship and variation tests, prevalence quantitative measures and double-variable tables (cross tabulation frequency); Relationship analysis: Pearson correlations and chi-square test of independence; Odds ratio were calculated after the variables were recoded into dichotomies and a multiple logistic regression model was used.

## Results

The first hypothesis claimed that female students present with a lower self and body image than males and engage in less physical exercise, yet keep a sensitive diet in higher frequency than male students. Table 1 presents the distribution of health perceptions, self image, body image, physical exercise and nutrition by gender. In total, approximately 40% of the students, with significantly higher frequencies (p<0.001) among females (47.2% females vs. 27.8 males) did not engage in physical exercise and only one third of the sample paid a lot of attention to eating a healthy diet. Almost half of the sample (47.8%) stated that they would like to lose weight and approximately one third reported that their health is much better compared to others their age. Males were found to have significantly higher perceptions regarding health, perceived their physical situation in a more positive manner, were more pleased with the way they look, had higher self image, kept a less balanced diet and performed more physical exercise, compared to females. Most of the comparisons shown in the table were meaningful and statistically significant.

**Table 1:** Distribution of variables by gender (%).

Measure	All % (SE) N = 1,565	Gender		Pearson Chi-Square
		Female% (SE) n = 1,036	Male% (SE) n = 529	
**Health perceptions**				
1. Feeling I am in good shape is very important	42.9 (±0.86)	37.7 (±0.87)	52.9 (±0.81)	***
2. Feeling I have plenty of energy is very important	60.0 (±0.68)	58.4 (±0.67)	62.8 (±0.71)	*
3. Keeping myself in good health all year round is very important	61.0 (±0.72)	59.4 (±0.72)	63.9 (±0.74)	
4. Keeping myself fit even if it takes some extra effort is very important	35.6 (±0.92)	31.3 (±0.92)	43.8 (±0.89)	***
5. My health is much better in comparison to others my age	35.5 (±0.81)	29.4 (±0.76)	48.4 (±0.86)	***
**Self Image**				
1. I make very good decisions about important things in my life	25.7 (±0.54)	25.4 (±0.54)	26.5 (±0.54)	
2. I am able to do well in school works	44.7 (±0.55)	43.7 (±0.54)	46.9 (±0.57)	
3. I handle setbacks and disappointments well	17.8 (±0.64)	14.7 (±0.63)	24.2 (±0.64)	***
4. On the whole, I am very satisfied with myself	20.4 (±0.56)	17.5 (±0.55)	26.5 (±0.57)	***
**Body Image**				
1. I think I am physically attractive to others	80.0 (±0.60)	80.4 (±0.60)	79.3 (±0.61)	
2. My physical shape is better in comparison to others my age	34.6 (±0.87)	29.2 (±0.82)	46.4 (±0.94)	***
3. I feel very satisfied with the way I look	22.1 (±0.61)	19.0 (±0.62)	28.4 (±0.55)	***
4. I would like to lose weight	47.8 (±0.86)	51.5 (±0.81)	41.0 (±0.93)	***
**Physical exercise**				
1. I engage in vigorous physical exercise (like running, riding a bike or lifting weights) more than 6 hours a week	10.9 (±1.09)	6.0 (±1.18)	20.7 (±0.40)	***
2. I do **not** engage in any physical exercise (like running, riding a bike or lifting weights)	40.6 (±1.09)	47.2 (±1.18)	27.8 (±0.40)	***
**Nutrition**				
1. I pay a lot of attention to ensuring my diet is healthy	33.2 (±0.72)	35.7 (±0.70)	28.0 (±0.75)	***
2. I pay a lot of attention to keeping down the amount of fat I eat	27.7 (±0.74)	30.0 (±0.72)	23.4 (±0.76)	***
3. I pay a lot of attention to eating some fresh vegetables everyday	44.7 (±0.72)	48.4 (±0.68)	37.5 (±0.76)	***
4. I pay a lot of attention to eating in a healthy way even when I eat out	26.3 (±0.71)	27.3 (±0.69)	24.1 (±0.74)	***
5. I pay a lot of attention to eating healthy snacks like fruit instead of candy	29.9 (±0.75)	32.7 (±0.76)	24.3 (±0.74)	**
6. I pay a lot of attention to eating baked or broiled food rather than fried food	28.1 (±0.73)	31.5 (±0.73)	21.5 (±0.73)	***
7. I usually snack instead of eating regular meals	15.4 (±0.66)	17.1 (±0.67)	12.0 (±0.65)	*
8. I skip breakfast most mornings	37.0 (±0.83)	36.9 (±0.84)	37.3 (±0.82)	

Pearson Chi-Square test of independence: *p<0.05,

** p<0.01,

*** p<0.001.

The second hypothesis claimed that positive relationships will be observed between engaging in physical exercise, healthy nutrition and positive self image and health perceptions. Table 2 presents the significant correlations (p<0.001) between health perceptions (variables 1–5) and eating habits (variables 6–13). All correlations were found to be significant in this test. Strong positive correlations were found between health perceptions and appropriate nutrition (correlations higher than 0.5 are marked). As expected, the stronger relationships were found among the content variables (between variables belonging to the same topic). Still, eating in a healthy way when eating out correlated with keeping in good health throughout the year (.307***) and keeping fit, even if it takes some extra effort (.308***).

**Table 2:** Significant correlations between health perceptions and the nutrition variables.

	1	2	3	4	5	6	7	8	9	10	11	12
1. Feel you are in good shape												
2. Feel like having plenty of energy	.602***											
3. Keeping in good health all year round	.535***	.578***										
4. Keeping fit even if it takes some extra effort	.744***	.526***	.553***									
5. Your health in comparison to others your age	.240***	.153***	.197***	.274***								
6. Your diet is healthy	.254***	.182***	.269***	.262***	.165***							
7. Keeping down the amount of fat	.264***	.191***	.291***	.326***	.166***	.671***						
8. Eating some fresh vegetables everyday	.183***	.140***	.243***	.214***	.178***	.496***	.484***					
9. Eating in a healthy way even when eating out	.251***	.200***	.307***	.308***	.207***	.564***	.561***	.536***				
10. Eating healthy snacks like fruit instead of candy	.242***	.199***	.281***	.286***	.184***	.549***	.527***	.559***	.619***			
11. Eating baked and steamed food and not fried food	.233***	.200***	.246***	.265***	.156***	.541***	.582***	.487***	.567***	.618***		
12. Snack instead of eating regular meals	.129***	.088***	.146***	.171***	.228***	.264***	.231***	.253***	.286***	.294***	.234***	
13. Skip breakfast most mornings	.134***	.085***	.144***	.147***	.143***	.301***	.260***	.271***	.291***	.278***	.235***	.286***

**Figure 1:**
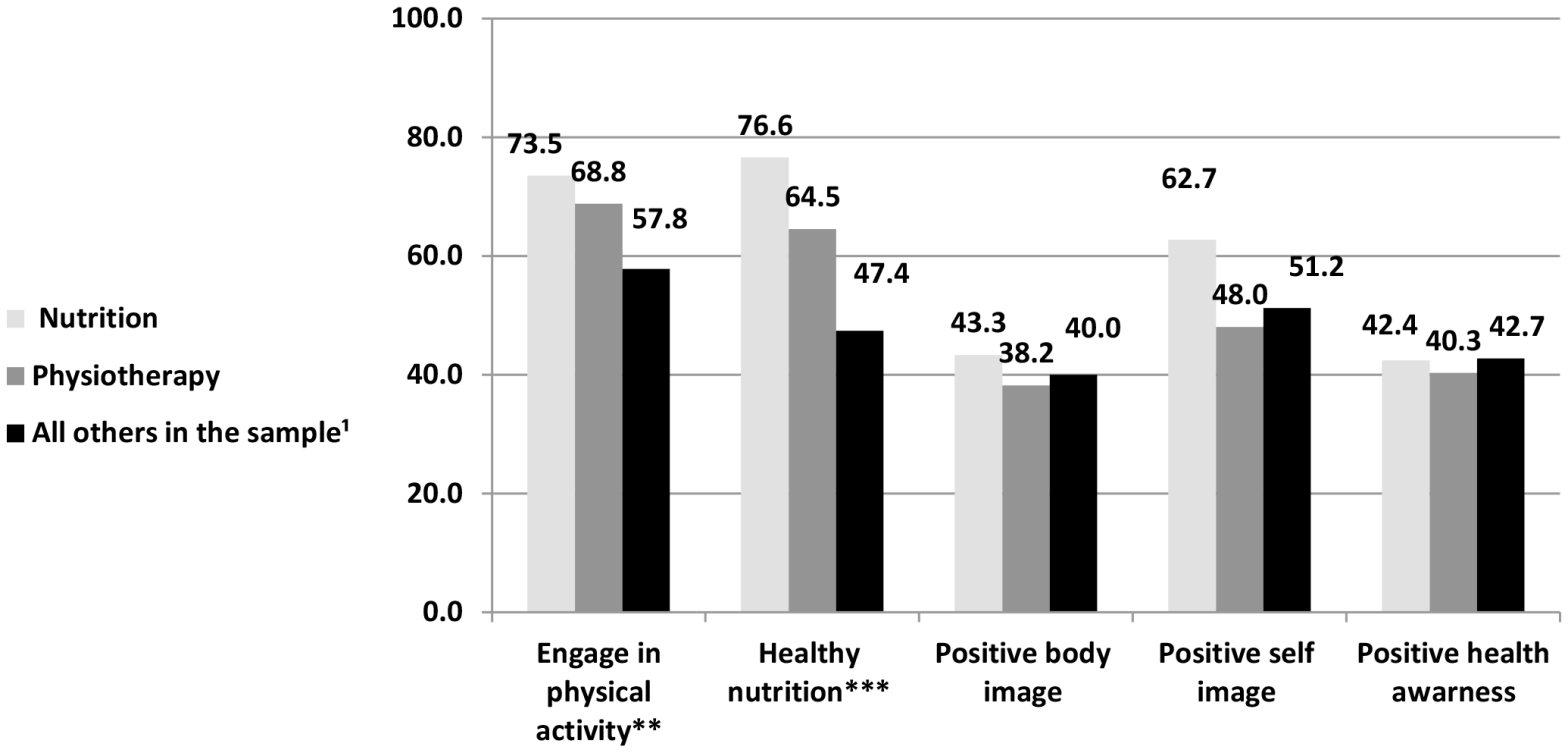
The distribution of various healthy lifestyle measures by field of academic study (%). N Total = 1,554 (Nutrition n = 69, Physiotherapy n = 125, All others in the sample n = 1,360). P<0.01**, p<0.001***.

According to the third hypothesis, nutrition and physiotherapy students will report improved healthy nutrition and physical exercise compared with students from other disciplines. The distribution of various healthy lifestyle measures among the different disciplines of study is presented in Figure 1. The field ‘All others’ refers to students from the following disciplines: health management, speech therapy, computer sciences & mathematics, molecular biology & biological chemistry, applicable & medical physics, economics & business administration, behavioral sciences, and multidisciplinary studies (humanities), not including students from the nutrition and physiotherapy departments. Indexes were created for health perceptions (5 items; α = .79), self image (4 items; α = .62), body image (3 items; α = .57) and nutrition (8 items; α = .85).

Our results indicate that nutrition students reported higher frequencies of positive health measures compared to students of all other disciplines, including physiotherapy. Engaging in vigorous physical activity (P<0.01) and maintaining healthy nutrition (p<0.001) were reported to be significantly higher among nutrition students (73.5% and 76.6% accordingly) than among physiotherapy students (68.8% and 64.5% respectively) and among the average student in the sample (57.8% and 47.4% respectively). Statistically significant differences were not found between the three study groups with regards to health perceptions, self image and body image.

Table 3 presents outcomes of a logistic regression model with significant odds ratios for healthy lifestyle. The table shows the unique contribution of each domain as a factor influencing other domains. Each domain is an outcome with the indexes of engage in physical exercises and healthy nutrition as predictors in the regression. We used regression in order to gain knowledge on what the most important variables may be. Engaging in vigorous physical activity predicted healthy nutrition (2.42, p<0.001), positive body image (2.38, p<0.001), positive self image (1.31, p<0.01) and positive health perceptions (3.01, p<0.001). Healthy nutrition predicted engaging in physical activity (2.42, p<0.001), and positive body image (1.79, p<0.001), positive self image (1.35, p<0.01) and positive health perceptions (2.52, p<0.001). The odds ratios for healthy nutrition and positive body image were more than twice as high (2.42 and 2.38 respectively) among the physically active students compared to non-physically active students. In addition, the odds of positive health perceptions among physically active students were more than three times higher (3.01) from those of non-physically active students. Nevertheless, students who reported maintaining healthy nutrition showed less meaningful odds for predicting positive body image (1.79), positive self image (1.35) and positive health perceptions (2.52). While the odds of 2.52 do indicate a meaningful outcome, they were still lower than the prediction odds of engaging in physical exercise with positive health perceptions (3.01). Therefore, the contribution of engaging in physical activity to positive body image and positive health perceptions was higher than the contribution of maintaining healthy nutrition.

**Table 3:** Odds ratio of healthy lifestyle measures logistic regression outcomes.

	Engage in physical exercises OR(CI)	Healthy nutrition index OR(CI)	Positive body image index OR(CI)	Positive self imageindex OR(CI)	Positive health perceptions index OR(CI)
**Engage in Physical exercises**	–	2.42*** (1.93, 3.03)	2.38*** (1.73, 3.27)	1.31** (1.06, 1.61)	3.01*** (2.20, 4.11)
**Healthy nutrition index**	2.42*** (1.93, 3.03)	–	1.79*** (1.30, 2.47)	1.35** (1.08, 1.68)	2.52*** (1.84, 3.46)

Odds ratio (Confidence interval of differences 95%) P<0.01**,

p<0.001***.

## Discussion

This study provides updated data on the association between self and body image and health perceptions among undergraduate students in a large academic institute in Israel. Overall, the results show considerable associations between health perceptions, appropriate nutrition, gender and study discipline.

Our first hypothesize suggested that female students present with a lower self and body image and engage in less physical exercise than males, although they keep a sensitive diet in higher frequency than male students. As expected, we found that males reported higher health perceptions, perceived their physical situation in a more positive manner, were more pleased with the way they looked, had a higher self image, kept less balanced diets and performed physical exercise at a higher frequency than females. This evidence is in line with previous studies which indicated that females reported significantly higher incidence of negative self and body image [15], [29], higher situational body dissatisfaction and higher rates of exercising for appearance-related reasons, [16], [17], [34].

Others have also found that females exercise less than males [35], and tend to keep a more balanced diet but are less aware or give a lesser value to health issues, compared to males [34]. This may be due to the fact that appearance is a more motivating value for women while health is more motivating for men. This difference can be understood through Self–Determination Theory (SDT) which describes personality development and self–motivated behavior change [36], [37]. According to this theory, people have an innate organizational tendency toward growth, integration of self, and resolution of psychological inconsistency. Of particular interest is how people internalize and integrate extrinsic motivations and come to self–regulate their behaviors in order to engage in daily actions in an autonomous manner. Autonomous regulation of behavior is considered to be both more stable and enduring, and to have more positive effects on human well being than controlled regulation [37]. Therefore, it may be that females attribute health more to nutrition than to sports and turn to nutrition in order to reduce caloric intake since they are less physically active and less pleased with their body image than males.

Previous studies have highlighted the stress resulting from academic requirements and additional difficulties of student life [23], [24]. While some have shown that female students tend to be more stressed than their male counterparts [21], others have shown that the inability to cope with stress and weight gain is correlated [25]. In this study, college students found their attempt to keep a healthy lifestyle or watch their weight during their studies to be more of an obstacle than an aid [10].

Our second hypothesis suggested that positive relationships will be observed between engaging in physical exercise, healthy nutrition, positive self image and health perceptions. As expected, strong positive correlations between health perceptions and proper nutrition were found, and health perceptions had a strong positive connection with a positive self and body image. Physically active students had higher health perceptions, and those with higher health perceptions also maintained better eating habits. Likewise, there appears to be a strong and steady connection between self image and body image variables. Our findings also show that the contribution of engaging in physical exercise to a positive body image and positive health perceptions is greater than maintaining healthy nutrition, which shows that students who engage in physical activity perceive their body and self image more positively than those who maintain healthy nutrition. Hence, for these students, physical activity has a greater impact on shaping positive self image than good nutrition.

The third hypothesis suggested that nutrition and physiotherapy students will report improved healthy nutrition and physical exercise compared with students from other disciplines. We found that nutrition students also reported higher frequencies of positive self and body image and engaged in more physical activity and appropriate nutrition than the average student in other academic disciplines, even more than among physiotherapy students, in contrast to our hypothesis. According to the literature, nutrition knowledge can help alter food choices [32] and nutrition students have more knowledge and obtain the foundation for conducting a healthy lifestyle as early as in their second study term, after they have already completed basic courses on nutrition, dietetics and food preparation, as well as courses demonstrating the relationship between physical activity, body weight, and the prevention and treatment of diseases.

Previous studies have demonstrated a strong need for health promotion in academic settings [34], [38]. This study suggests, as have others before, that successful health promotion policy should reflect a collectivist rather than an individualist ethos by providing the prerequisites of health through health-promoting public policy, where academic settings support a healthy lifestyle policy by increasing availability of a healthy, nutritious and varied menu in cafeterias, and offering students activities that enhance healthy eating and exercise. This health promoting policy should start at the top, with key teaching professionals in the facility, in order to ensure they receive proper training about nutrition, eating disorders, weight control, and suitable tools to better advise overweight students, or where to refer them so that they can obtain this information themselves.

Moreover, a system for early identification of risky health behaviors is essential for the design of health interventions since targeting specific student needs can help administrators, curriculum planners, and community health professionals design guidelines for structuring a healthier environment and developing health education programs that support healthy choices among students [9], [38]. One study on worksite weight management interventions showed that such interventions have the potential to reduce sick leaves, health care costs and workers compensation costs, as well as to increase moral and efficiency [39]. These findings could potentially be translated into academic settings to help students obtain all of these potential benefits and thus enhance their academic achievements and satisfaction. Nevertheless, at-risk individuals may benefit more from individual counseling programs [34], and the plausibility of providing such programs on campus should be explored.

These findings are constrained by the study’s four main limitations. First, this study is based on cross-sectional data. There is considerable self-selection going on when people decide their field of study, for example nutrition. It could well be that the people with higher levels of these preexisting variables were more likely to choose these fields, and so it is difficult to determine whether the study could clearly examine its hypothesis with the current design. However, the descriptive data found in our study is interesting and greatly contributes to existing scientific knowledge, yet with some limitations regarding cause-effect relationships. The second, is that this sample is based on one local university campus. The third limitation stems from the fact that the measures in this study are based on self-reports. Finally, we constructed the body and self image measures with a Cronbach's Alpha which is typically considered to be low, by using Jessor's questionnaire guide, in which he reported alpha  = .71 for the 8 self esteem items. In our study self and body image were found to be interesting as two different domains, therefore, we separated questions referring to each domain specifically, an action which may further lower the Cronbach's Alpha. Future studies should take this into account and use additional well-validated measures.

### Conclusions

Academic settings should use health-promoting public policy to increase the availability of healthy, nutritious and varied menus in cafeterias, offer students various activities that enhance healthy eating and exercise, and develop screening and support systems for those at risk. In addition, physical education classes should be a mandatory prerequisite course for the completion of an undergraduate degree, as long as the foundation for enabling students to engage in physical activity and encouraging it are available within the academic settings.
